# Are Autumn Foliage Colors Red Signals to Aphids?

**DOI:** 10.1371/journal.pbio.0050187

**Published:** 2007-08-14

**Authors:** Lars Chittka, Thomas F Döring

## Abstract

Why do plants change color in the autumn? Could it be a signal to aphids, warning them of the defensive strength of the trees, or might the cause be more mundane?

*“…bright colours of leaves in the autumn are a warning signal to insects that lay their eggs on the trees in that season.” —Marco Archetti and Sam P. Brown (2003) [1]*

*“The assumed attractiveness of bright colours to insects would appear to involve the supposition that the colour vision of insects is approximately the same as our own. Surely this is a good deal to take for granted.” —Lord Rayleigh (1874) [2]*

*“Autumn colouring is of great interest in a comparative study of coloration. There is no reason to suppose that the colouring is of the slightest use to the trees, and yet it often displays to an extraordinary degree that beauty and perfectness which we are accustomed to regard as the result of the action of Natural Selection.” —Marion I. Newbigin (1898) [3]*

## Introduction

Most living things don't turn beautiful when they senesce. Aging flowers, for example, typically become wrinkly and tattered, and their colors become dull. The leaves of many temperate tree species, on the other hand, display veritable fireworks of colors—just before they fall to the ground and rot. We know that some of the pigments causing this coloration simply become unmasked when chlorophyll disintegrates, while others protect leaves from the combined detrimental influences of cold and intense sunlight. Two studies at the beginning of this decade, however, suggested a radically different explanation: that “bright” autumn colors might in fact be signals to aphids, warning them of the defensive strength of the trees that produce them. This suggestion has prompted a fascinating debate between plant physiologists (most of whom believe that the pigmentation serves physiological processes inside the leaves), and some evolutionary theoreticians (who conjecture that such exuberant colors must serve a signaling function). Colors, however, are not simply manifestations of physics—they are generated by animals' brains, depending on the particular sensory apparatus the viewers have acquired in their evolutionary history. Insect color receptors and post-receptor neuronal processing are so fundamentally different from those of humans that what appears bright to us can sometimes be cryptic for them, and what we view as a deterrent might in fact be attractive to insect herbivores. We take a look into the fascinating color world of aphids, and conclude that, while aphids clearly respond to color signals, our current knowledge does not support the notion that autumn tree colors could be an efficient means to deter aphids.

## Physiological Functions of Autumn Leaf Pigments

**Figure 1:**
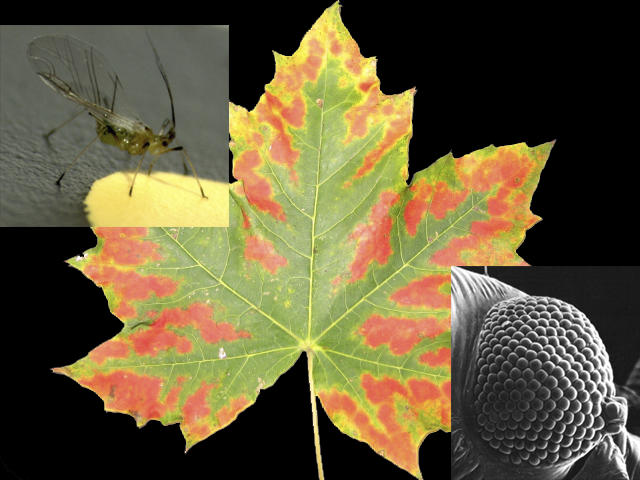
Autumn Tree Colors as Warning Signals to Aphids? Two papers at the beginning at this decade challenged our view that the beauty of autumn leaves is only a by-product of physiological processes inside the doomed leaves [13,14]. According to the new hypothesis, trees with particularly strong coloration send an honest signal to aphids, informing them of the strength of anti-herbivore defenses of these trees. But to appropriately predict the responses of aphids to colors requires us not only to examine the physiology of their eyes (inset lower right: scanning electron micrograph of the eye of the black bean aphid Aphis fabae, courtesy of J. Hardie) but also their behavioral responses to colors under controlled laboratory conditions (inset, upper left: the foxglove aphid Aulacorthum solani probing a yellow artificial target; photo by S. Kirchner).

Leaves are the biomaterials factories of trees. However, deciduous trees need to close down these factories in preparation for winter: temperatures are too low for photosynthesis, and leaves would lose large quantities of water through evaporation that could not be replenished from frozen soil [4]—plus there is a risk of tissue damage through freezing [5]. Yet leaves do not simply rot until they fall off the tree: in fact, the metabolic rate in trees sometimes increases before abscission [6]. This increase occurs because leaves often continue to be photosynthetically active, while at the same time, trees scramble to recover large amounts of nutrients from the leaves before shedding them—up to 60% of dry mass [7] and up to 70% of nitrogen [8]. But why would leaves unleash such potentially wasteful riots of color during this recovery operation (Figure 1)?

In school, we were taught that these colors are mere by-products of disintegration. The red anthocyanines, however, are specifically manufactured during autumn [3], and their physiological roles are so diverse that they have been called “Nature's Swiss army knife” [9]: they are powerful antioxidants and protect against a process called photoinhibition, where a combination of low temperature and strong sunlight impairs a key biochemical process in photosynthesis [9]. It has also been suggested that they might act to protect the planned deconstruction of the photosynthetic machinery, function as sinks for harmful substances such as heavy metals, warm the leaves, and protect against harmful UV (ultraviolet)-B radiation [9–11].

## Is Beauty in the Eye of the Beholder?

But in addition to these suggested roles, do autumnal leaves also communicate to animals? M. I. Newbigin (1869–1934), a remarkable woman whose extraordinary vision in presiding over the Scottish Geographical Magazine is deemed unrivalled among editors of scientific journals of the time [12], pointed out eloquently that one should resist the temptation to attribute signaling function to everything that's colorful (see [3] above and at end of article). The yellow of egg-yolks or the orange of carrots are obvious examples. Nonetheless, a paper published posthumously by one of the 20th century's most influential biologists, W. D. Hamilton, and co-authored by S. P. Brown, and separately, the first publication by a young evolutionary theoretician, M. Archetti, suggested a function that departs radically from what the textbooks tell us about autumn tree coloration: bright autumn tree colors might be warning signals to aphids (and other herbivores), to deter them from settling on the leaves in the autumn [13,14]. They propose that plants “honestly” signal their defensive strength to aphids, so that more brightly colored individuals are less palatable; and thus signal receivers had better heed the warning [13,14].

**Figure 2:**
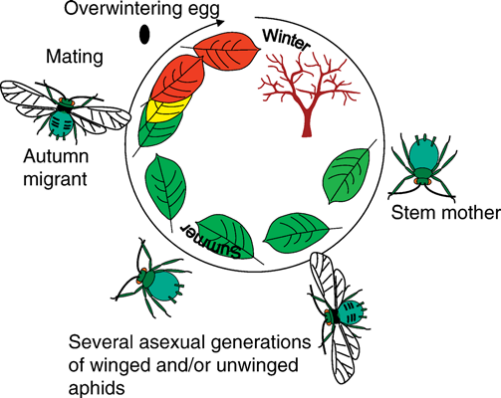
Typical Aphid Annual Life Cycle In spring the stem mother (fundatrix) hatches from a fertilized egg and asexually produces female offspring. During spring and summer, there is little variation between target colors, since most leaves will be green. In autumn, winged aphids migrate towards their winter hosts, and might choose between leaves based on variation in color cues. Note that many aphid species diverge considerably from this stereotypic life cycle.

This suggestion is not as far-fetched as it may seem. Despite their insignificant appearance, aphids can severely affect the health of trees, both by direct consumption of plant material and through transmission of viruses [15]. Up to several dozen aphids may feast on a single leaf, and they have a powerful reproductive potential, since for much of the year, they don't waste time on sex or egg-laying: mothers clone themselves, and can give birth to live daughters that are already pregnant when born [15]. Based on observations by French entomologist and military commander René A. F. de Réaumur (1683–1757) [16], it has been estimated that a single aphid could produce 5.9 billion offspring in six weeks [15], and undoubtedly such numbers indicate that aphid colonization can be damaging for plants. Sycamore trees, for example, might produce 280% more stem wood if aphids were removed [15], and bud size and fruit set can be severely affected by heavy aphid infestation [17]. Therefore, trees could strongly benefit by finding a suitable signal to keep aphids from alighting. But why would it be useful to repel aphids in the final few days before the leaves are shed? In autumn, legions of winged aphids take to the air, find their suitable winter hosts, mate, and lay eggs on tree branches (Figure 2) [15]. Thus, a tree that successfully warns off aphids at this time of the year might spend the early months of the subsequent year undisturbed.

Hamilton and Brown [14] posit that trees would have to be honest about their palatability and their chemical anti-herbivore defenses, because they simply couldn't afford to lie. They suggest that trees act on the same principle as the male peacock and the gold chain–wearing boys in the discotheque: if you are bearing costly signals, then you must have the means to produce them, and this ensures that the signal is a reliable source of information for the receiver [18]. So goes the theory.

In fact, however, some of the pigments that we come to see in autumn have been there all along. The yellow xanthophylls (a type of carotenoid) are an integral part of photosynthesis, and therefore occur in all green leaves [19]. These pigments simply become unmasked when chlorophyll disintegrates [3,20]. Yellow pigments in autumn leaves cost the plant nothing—hence the suggestion that only trees with stronger chemical anti-herbivore defenses can afford to produce more “yellowness” is not well supported. Red anthocyanines, on the other hand, are produced specifically during autumn—but as explained above, they serve multiple physiological functions [9,21], and the cost might often be marginal [22].

## What Is Known about Aphids' Color Perception?

**Figure 3:**
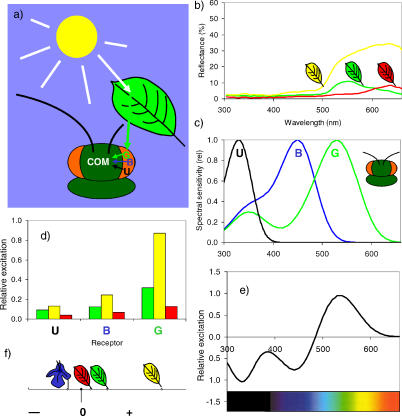
The Perception of Colored Leaves by an Aphid (A) A green leaf reflects the light from the sun and is seen by an aphid with a green (G), blue (B), and ultraviolet (U) photoreceptor [29]. Evidence from behavioral studies [31] indicates that a COM processes the input from the photoreceptors. (B) Reflectance spectra of three leaves from the bird-cherry, Prunus padus. To humans, these leaves appear green, red, and yellow. For measurement methods see [26]. (C) Tentative spectral sensitivities of the green peach aphid's UV, blue, and green photoreceptors. (D) Relative excitation of the UV, blue, and green photoreceptors produced by the green, yellow, and red bird-cherry leaves shown in (B). For methods of calculation, see [48]. (E) Excitation spectrum of a COM inferred from behavioral data [26,31]. The mechanism is fed by the three photoreceptors in (C), with positive input from the green and negative input from the other two receptors. Based on behavioral data, a mechanism of this kind is presumably what many insect herbivores use as a “greenness detector.” (F) Excitation of the COM by the three bird-cherry leaves and a blue-purple flower (Ajuga genevensis) on a one-dimensional scale. Note how the yellow leaf produces an even more positive signal than the green leaf.

A further complication with the autumn signaling hypothesis is that it is at odds with a large body of literature on herbivorous insects' visual systems, and their behavioral responses to color signals. More than a century and a quarter ago, Physics Nobel laureate Lord Rayleigh mocked the view that what is bright to human observers should also be bright to insects [2]. Still in the 19th century, the first empirical support for fundamental differences between human and insect vision was found by John Lubbock (later Baron Avebury)—a banker and a member of the British Parliament who expressed regret that his parliamentary duties would sometimes keep him from his entomological research [23]. He introduced bank holidays and discovered that ants perceive UV light [24]. In the next century, studies were conducted on the wavelength sensitivities of insect eyes of dozens of species, including many that feed on leaves, as well as on their behavioral responses to colors [25,26]. At present, it appears that the eyes of all herbivorous species so far tested, including aphids, locusts, potato beetles, and herbivorous caterpillars [26–29], contain three types of color receptors, each maximally sensitive in the UV, blue, or green spectral domain (Figure 3). No herbivorous insect studied to date has red color receptors in its eye as humans do [26], although many other insect species do possess such receptors [25], and it would be necessary to test more herbivores before reaching a conclusion.

To calculate the receptor signal that is produced when a photoreceptor views a particular target, one needs to calculate the area of overlap (integral) between the spectral power distribution of the illuminating light (Figure 3A), the target's spectral reflectance function (Figure 3B), and the receptor's spectral sensitivity curve (Figure 3C). In the green peach aphid (Myzus persicae), the only species studied thoroughly to date, extracellular electrophysiological recordings were performed by placing an electrode into the eye of a tethered aphid, and recording the change in voltage potential as a result of stimulation with different wavelengths. These recordings revealed the existence of a UV receptor with maximum sensitivity near 330 nm and a green receptor maximally sensitive at 530 nm [29]. Based on these peak sensitivities, precise sensitivity functions can be modeled for each individual photoreceptor type [30]. Additionally, the existence of a third, blue receptor is certain in the green peach aphid, but its precise position of maximum wavelength sensitivity remains to be determined [29]. In the absence of evidence to the contrary, we have here placed the blue receptor in the position where it most commonly occurs in the pterygote insects (Figure 3C) [25]. Using this information, the receptor's graded voltage potentials can be calculated (Figure 3D).

But how are these receptor potentials further processed, and how do they drive aphid behavioral responses to colors? A behavioral experiment by an eminent aphid biologist, V. Moericke, offers insight into the neural processing of color by these tiny insects [31]. Under controlled laboratory conditions, green peach aphids will enthusiastically try to drill their proboscides into surfaces that reflect green to yellow light. However, these aphids would largely ignore red or blue surfaces, or grey ones. This is consistent with the notion that the aphid's green receptor contributes an excitatory input to the motor pattern of proboscis extension [26]. But when Moericke presented the aphids with grey surfaces immediately after they had been viewing a blue or violet stimulus, the aphids suddenly found the grey target worth probing. The surprising change in their response to a grey (neutral) target is reminiscent of successive contrast phenomena in humans: when you fixate a violet target for 30 seconds, and then stare at a white surface, you briefly see yellow [32]. Although the physical properties of the surface had not changed, the aphids saw the illusion of a color that they found attractive. This phenomenon is based on two processes, receptor adaptation and color opponency. Adaptation makes a photoreceptor more sensitive when there is little light in its spectral domain for an extended period, and it makes a receptor less sensitive when it is strongly stimulated [33]. Staring at a violet/blue surface makes an insect's (or human's) short wavelength receptors less sensitive, and the long wavelength receptors (the aphids' green receptors) more sensitive. When the aphid subsequently encounters a spectrally neutral surface, it will view it with highly sensitive green receptors (which will thus send a stronger signal to the aphid brain) and less sensitive short wave receptors. However, for the grey surface to appear more attractive, there must be a neural comparison between input from the green receptor and those from the shorter wavelength receptors —in other words, a spectrally opponent mechanism ([26]; Figure 3E). In aphids, evidence for receptor adaptation and spectral opponency can only be inferred indirectly from behavioral tests. However, adaptation is an inherent property of all photoreceptors [33], and color opponent neurons have been found in several insects [25,34]. Successive contrast phenomena have also been found in other insects [35]. Hence it is parsimonious to postulate a color opponent mechanism (COM) of the type displayed in Figure 3E in the green peach aphid. Although the precise excitation spectrum of the COM is still hypothetical, the general type of mechanism, with a green versus short wavelength component, is backed by a multitude of studies on the behavioral response of aphids to colors [26].

## Behavioural Responses of Aphids to Autumn Leaf Colors

With such a mechanism as a foundation, it becomes possible to explain the color preferences of the green peach aphid (and many other species of aphids [26]) concisely in mechanistic terms. When the excitation of the COM by three bird-cherry (Prunus padus) leaves (and, for comparison, by the blue-purple flower Ajuga genevensis) is displayed on a one-dimensional “greenness” scale, the red leaf is, in fact, less stimulating for this mechanism than the green leaf (Figure 3F). Thus, while red and yellow might both appear bright to humans, most red leaves are predicted to appear relatively dull and cryptic (albeit not invisible! [36]) to the herbivorous insects so far tested ([26]; Figure 3F). To measure crypsis or conspicuousness quantitatively, spectral reflectance measurements are necessary [26,37], but it is unlikely that most shades of leaf-red could serve as an efficient deterrent for these insects.

In many herbivorous insects, the response to yellow targets is fundamentally different than the response to red, and wholly at odds with the hypothesis that yellow could work as a general repellent to insects. Insects in search for leaves often have a preference for the color green [38]—in other words, for targets that stimulate their green receptors more than their UV and blue receptors. However, yellow targets with their often high-intensity reflectance stimulate the green receptor even more strongly than green foliage—especially in comparison with the low excitation of the UV and blue receptors (Figure 3). In insects with a color opponent mechanism that pits the response of the green receptor against that of the UV and blue receptor, yellow produces a particularly strong signal (Figure 3F), and thus serves as a powerful attractant to many insects that are actually looking for green [26,39]. Hence, yellow has been termed a “supernormal foliage-type stimulus” [38] that is more attractive to most species of aphids than green [26,31]. Correspondingly, yellow insect traps are highly effective in monitoring many species of aphids [26]. If trees wanted to deter herbivorous insects using color, yellow leaf coloration is about the worst strategy they could pick.

Anecdotal evidence from field studies supports the predictions that yellow leaves are attractive for aphids [22,40], whereas red can be insufficiently distinct from green to provoke a response. California maple aphids (Periphyllus californiensis) appear to ignore red-leaved Japanese maples, but happily settle on yellow-orange ones [41]. In a study with dyed leaves, red leaves appear to be no different from green ones in terms of attractiveness to landing aphids [42]. Hamilton and Brown [14] found that the more “yellowness” a tree species displayed, the more aphid species colonized the trees. This was taken as evidence that trees under pressure from aphids need to invest more into repellent signaling—but the reverse may actually be true: the very reason why those trees are attractive to more aphid species could be their palatability and the absence of suitable defenses. Thus, Hamilton and Brown's own observations support the notion that yellow foliage coloration conveys, if anything, a disadvantage that might bring in more aphids than would alight if the leaves stayed green until abscission. There are some species of aphids that prefer green over yellow natural leaves, however [43].

## The Need for More Laboratory Studies

The problem with such observational and correlational studies, however, is that leaves that differ in color might also differ in texture, chemosensory cues (both taste and scent), nutrient content, toxicity, texture, and temperature—and insects respond to all of these cues [44–47]. We need to take these studies into the laboratory, and expose insect herbivores to targets that are identical in all the above parameters but color. Scoring colors simply by brightness today is no less hazardous than it was in Lord Rayleigh's day—spectral reflectance measurements (including UV) are needed (Figure 3) [48]. To assess the significance of any biological color signal, we need data on spectral sensitivities of the receiver visual system, as well as information about post-receptor neuronal wiring [26,49]. In the most-studied species, the green peach aphid, there is good behavioral evidence for a spectrally opponent mechanism that compares input from the green receptor with that from the shorter wavelength receptors. As a result, and perhaps surprisingly for human observers, some shades of red are more similar to leaf green than yellow is to either red or green (to a peach aphid). Note that any other neural method of evaluating the information from the receptors—such as a two-dimensional color opponent system as in bees [49], or a strictly categorical system as in blowflies [50]—might produce fundamentally different relative perceived similarities between these colors. There is no way of “guessing,” even approximately, the conspicuousness of a target for an animal by using human visual assessment, or an in vacuo measurement of UV signals [51]—we need data on receptor spectral sensitivities as well as post-receptor neural processing, either from neurophysiological tests or carefully designed psychophysical tests.

Clearly, there is also an urgent need for data from more species of aphids—the desirable combination of solid behavioral and physiological data are currently only available for one species (see above), but from several other species so far tested, there is clear behavioral evidence that “bright” autumn coloration may either be maladaptive (yellow) or neutral (red) for protection against aphids [26]. There are some aphid species, however, that indeed appear to prefer green over yellow [26,43], so a thorough comparative study might reveal interesting differences between species, depending on their ecology and their particular relationship to autumn foliage coloration.

The autumn tree color hypothesis is a perfect example of how looking at color signals through the eyes of humans rather than the potentially intended receivers may have sometimes led scientists astray. Insufficient knowledge of the receiver system could cause us to mistake conspicuousness for crypsis, or deterrents for attractants. At present, it appears that multiple established functions of yellow carotenoids (e.g., as integral components of photosynthesis, and protectors against photo-oxidative damage [19]) and red anthocyanines (e.g., as free radical scavengers and protectors against photoinhibition [9]) seem perfectly sufficient as explanations of why trees turn colorful in the autumn. We leave the reader with a paragraph from M. I. Newbigin's monograph *Colour in Nature* [3]. While this statement is a caveat against hasty acceptance of adaptationist hypotheses about color in animal signaling, it is just as pertinent to plant pigmentation:

*“Thus, ... colour, wherever seen, is due to the favouring influence of Natural Selection, and is in some way useful to the species. In view of the popularisers of the subject, it therefore becomes the main object of the naturalist to invent as ingenious an explanation as possible of the way in which it is useful. If the naturalist's powers of invention fail, though this happens but rarely, then the colour is non-significant, or better still, the animal has recently changed environment, and is no longer perfectly adapted to its environment. The theory is, therefore, perfectly complete and coherent, and persons refusing to accept it are at once stigmatised as laboratory-made scientists, ignorant of nature, and unworthy of the name of naturalist.” —M. I. Newbigin (1898) [3]*

## Abbreviations

COM: color opponent mechanism
UV: ultraviolet
